# An automated fitting procedure and software for dose-response curves with multiphasic features

**DOI:** 10.1038/srep14701

**Published:** 2015-10-01

**Authors:** Giovanni Y. Di Veroli, Chiara Fornari, Ian Goldlust, Graham Mills, Siang Boon Koh, Jo L Bramhall, Frances M. Richards, Duncan I. Jodrell

**Affiliations:** 1 CRUK Cambridge Institute, University of Cambridge, UK; 2 NIH Chemical Genomics Center, National Institutes of Health, Bethesda, USA

## Abstract

In cancer pharmacology (and many other areas), most dose-response curves are satisfactorily described by a classical Hill equation (i.e. 4 parameters logistical). Nevertheless, there are instances where the marked presence of more than one point of inflection, or the presence of combined agonist and antagonist effects, prevents straight-forward modelling of the data via a standard Hill equation. Here we propose a modified model and automated fitting procedure to describe dose-response curves with multiphasic features. The resulting general model enables interpreting each phase of the dose-response as an independent dose-dependent process. We developed an algorithm which automatically generates and ranks dose-response models with varying degrees of multiphasic features. The algorithm was implemented in new freely available *Dr Fit* software (sourceforge.net/projects/drfit/). We show how our approach is successful in describing dose-response curves with multiphasic features. Additionally, we analysed a large cancer cell viability screen involving 11650 dose-response curves. Based on our algorithm, we found that 28% of cases were better described by a multiphasic model than by the Hill model. We thus provide a robust approach to fit dose-response curves with various degrees of complexity, which, together with the provided software implementation, should enable a wide audience to easily process their own data.

Measuring drug effects on biological systems is part of many scientists’ routine^1^,^2^. Observed effects span from the inhibition or agonism of proteins and other molecules^3^,^4^ to effects measured at the cell^5^, tissue^6^ or whole organism levels^7^,^8^. In cancer research, cell proliferation and viability are often assessed in a panel of cell lines specific to a given type of cancer^9^. Typically, the biologist or pharmacologist compares populations of treated vs. untreated cells at various drug concentrations. The data is summarized via a dose response curve and then fitted using an in-house program or commercial software. The fitted curve gives a mathematical description of measured effects and enables interpolating or extrapolating missing information. When various cell lines or drugs are also investigated, the resulting models facilitate comparing dose-responses by summarizing them via a few parameters^10^ (e.g the relative 50% effective concentration *EC*_*50*_).

The fitting procedure that follows data acquisition can be challenging from several point of views. First of all, for most experimentalists who are not familiar with modelling, this procedure will require the availability of friendly software to fit the data. Moreover, most available programs will attempt to fit the data to a standard Hill model (also called 4 parameters logistic^11^). The Hill model has been used extensively in the past. It can describe chemical reactions in mechanistic terms^12^ and enables excellent modelling of most cases. However, the Hill model is based on a unique point of inflection and cannot faithfully describe cases where agonist (stimulatory or hormetic) effects are also observed^13^,^14^. Moreover, even in the absence of agonist effects, cases where there is more than one point of inflection in the inhibitory phase cannot be handled^15^. This can result in poorly fitted curves which can mislead data interpretation, comparison to other cases, and extrapolation if such fits are accepted. Recently, Haibe-Kains *et al.*^16^ identified the choice of an estimator for summarising drug dose-response curves as one of the reasons for lack of correlation of drug sensitivity in large pharmacogenomics screens^17^,^18^. At the same time, the importance and implications of multiphasic dose-responses have been highlighted in various contexts^19^,^20^ including cancer therapeutics^21^,^22^.

Extensions of the Hill model for asymmetrical curves have been developed, but these do not accommodate several points of inflection^23^,^24^,^25^,^26^. Alternatives have been suggested to handle biphasic dose-response curves^27^,^28^. These were tailored to the specific case of an initial stimulatory effect and are not necessarily equipped with a common structure and straightforward interpretation of the resulting model. There have also been a number of efforts in improving dose-response fitting^29^,^30^ and several free software products are available to fit dose-response curves^31^,^32^,^33^,^34^. These efforts often focused on the standard Hill model and did not provide a free tool which can be used to generate multiphasic dose-response models in a user-friendly manner as per well-known commercial packages^35^,^36^,^37^. Note that there also exists general free language and environment such as R^38^ which easily enable cubic splines fitting (splines are curves generates by connecting polynomials). Nevertheless, splines fitting is not optimal due to the lack of model structure which affects the goodness of fit and prevents interpretation in mechanistic terms.

Here we present a general model which enables excellent fitting of dose-response curves with multiphasic features. From a theoretical point of view, this new model combines dependent, cooperative effects as described by a Hill model, with independent effects as suggested by the Bliss approach for combination studies^39^. We show that non-regular cases encountered in cancer pharmacology can be satisfactorily handled by this approach which combines these two classical pharmacological models. We have developed an algorithm which enables automated fitting of dose-response curves and have implemented it in freely available software (*Dr-fit* as per **D**ose-**r**esponse **Fit**ting). This approach was successful in modelling dose-responses which could not be described by a standard Hill equation. We then analysed a large screen involving 11650 dose-response curves and found that a substantial proportion of cases were better described by this approach.

## Results

### From Hill to multiphasic models

**Figure 1:**
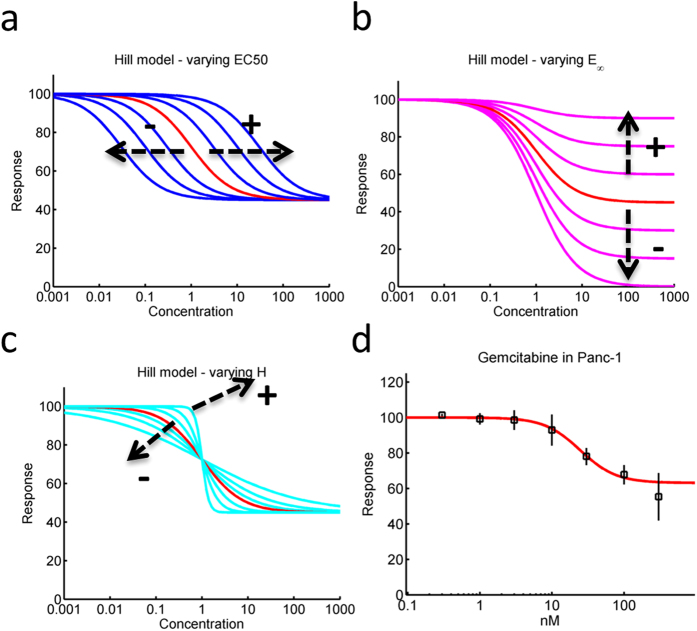
Standard Hill model. **(a)** The Hill model enables generating sigmoidal dose-response curves. It can be shifted in concentration space by varying its EC_50_ parameter. **(b)** The E_∞_ parameter (also called E_max_) can be used to modulate effects at high concentration. **(c)** The Hill coefficient modulates the slope of the curve. **(d)** The flexibility of this model captures dose-response data in most cases (here an example of Gemcitabine in the Panc-1 cell line is shown).

The Hill model is based on the following equation which describes the effect *E* obtained at a given concentration *C*:


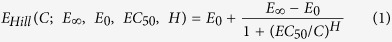


where *EC*_*50*_ is the relative 50% effective concentration, *H* is the hill exponent, *E*_*∞*_ is the maximum effect and *E*_*0*_ is the effect in the absence of drug. This equation can also be manipulated and written under alternative forms or via different definitions of its parameters. If the dose response is built by considering a measure of the system being studied (e.g. amount of cells or of proteins) in treated conditions over this same measure in untreated conditions, then the baseline value *E*_*0*_ is fixed to unity (the dose-response can also be expressed in terms of percentage as it is done here). Figure 1a shows the typical sigmoidal curve that is obtained with the Hill model. The figure also shows that varying the *EC*_*50*_ shifts the curve in log-space while varying the *E*_*∞*_ changes the effect level obtained at high concentrations (Fig. 1b). Finally, the hill exponent *H* can be used to account for various degrees of steepness (Fig. 1c). This model can therefore be used to fit typical dose-response curves encountered in pharmacological studies (Fig. 1d).

**Figure 2:**
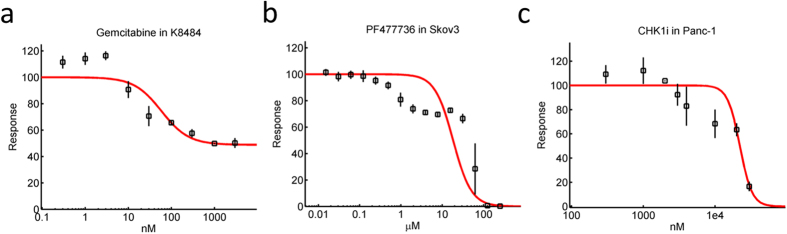
Non-monophasic cases. **(a)** In a number of instances, observed dose-responses are not monophasic. In this example (Gemcitabine in the K8484 cell line), an initial stimulatory effect can be observed. (**b)** There are also instances where two points of inflection are observed in a dose-response with purely inhibitory features (here an example of PF477736 in the Skov3 cell-line is shown). **(c)** In some cases, both the presence of an initial stimulatory effect and two phases in the inhibitory range can be observed (here an example of CHK1i in the Panc-1 cell-line is shown). In all these cases, the dose-response cannot be captured with the optimization of a standard Hill model (red lines).

In a significant number of cases, dose-response curves show stimulatory effects (notably at low concentration; Fig. 2a), or two point of inflections (Fig. 2b), or even a combination of these features (Fig. 2c). In these cases, it is obvious that attempting to fit the data to a Hill model cannot result in a satisfactory description of the data (red lines in Fig. 2a–c). Here we propose a modelling approach that is based on breaking down each one of the observed phases into independent, separate processes. Then each of these distinct processes is considered as the observed effect of closely related sub-processes. The mathematical formulation of this approach is as follows.

We first consider each phase separately and model it using a standard Hill model. For each phase *i* we write:


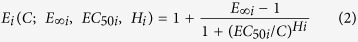


Then we consider each one of these phases as being part of successive reactions which independently converge toward the same phenotype, thus resulting in the total effect E:


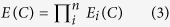


where E(C) is the observed dose-response curve, and E_i_(C) is the dose response curve corresponding to the underlying ith independent process. Note the well-known similarity between this formulation and the probability of independent events. This leads to the following model when considering all phases:


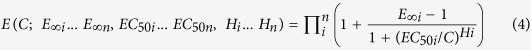


**Figure 3:**
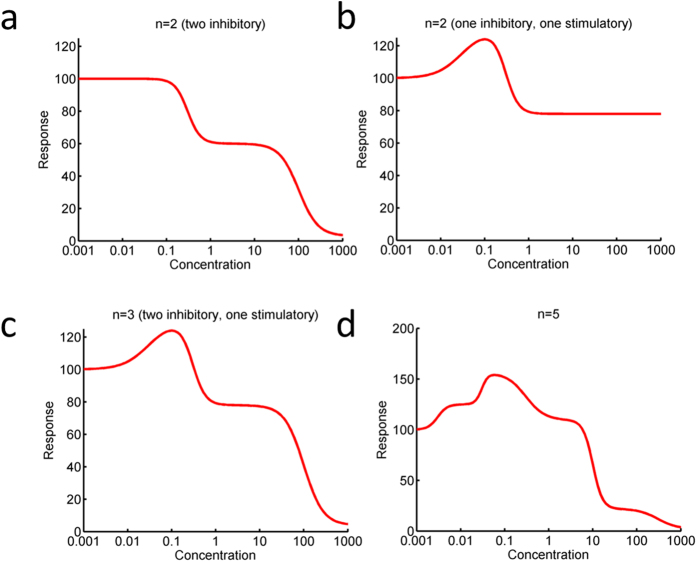
New general multiphasic model. Our model enables description of a variety of dose-response cases where various phases are present. Simulated examples are shown for hypothetical cases with **(a)** two inhibitory phases, **(b)** one stimulatory and one inhibitory phase, **(c)** a combination of a stimulatory and two inhibitory phases. **(d)** A complex example involving two stimulatory and three inhibitory phases.

Note that this formulation is only correct when the response in the absence of drug is unity. Alternative formats can nevertheless be obtained by scaling this equation appropriately. Using this model with n = 2 two phases of inhibition (Fig. 3a) or one stimulatory and one inhibitory phase (Fig. 3b) can be described. Using n = 3, a multiphasic dose response with one stimulatory and two inhibitory phases can be described (Fig. 3c). There is no theoretical limit and for instance using n = 5, we can also describe more complex dose-response curves such as one that involves two stimulatory and three inhibitory phases (Fig. 3d).

It is important to realize that the higher the number of process incorporated (i.e. the greater is n), the more it is difficult to experimentally discern the presence of each process due to inherent biological variation and experimental error. Also, the inclusion of each additional phase requires 3 parameters, so for n = 3 phases, 9 parameters are required. Therefore we compared the classical mono-phasic case (Hill equation) with three configurations only. These were cases where two stimulatory (n = 2), or one stimulatory and one inhibitory (n = 2), or one stimulatory and two inhibitory phases (n = 3) were present. In our experience, these configurations enable to describe most cases encountered in cancer pharmacology.

### Automated curve fitting

**Figure 4:**
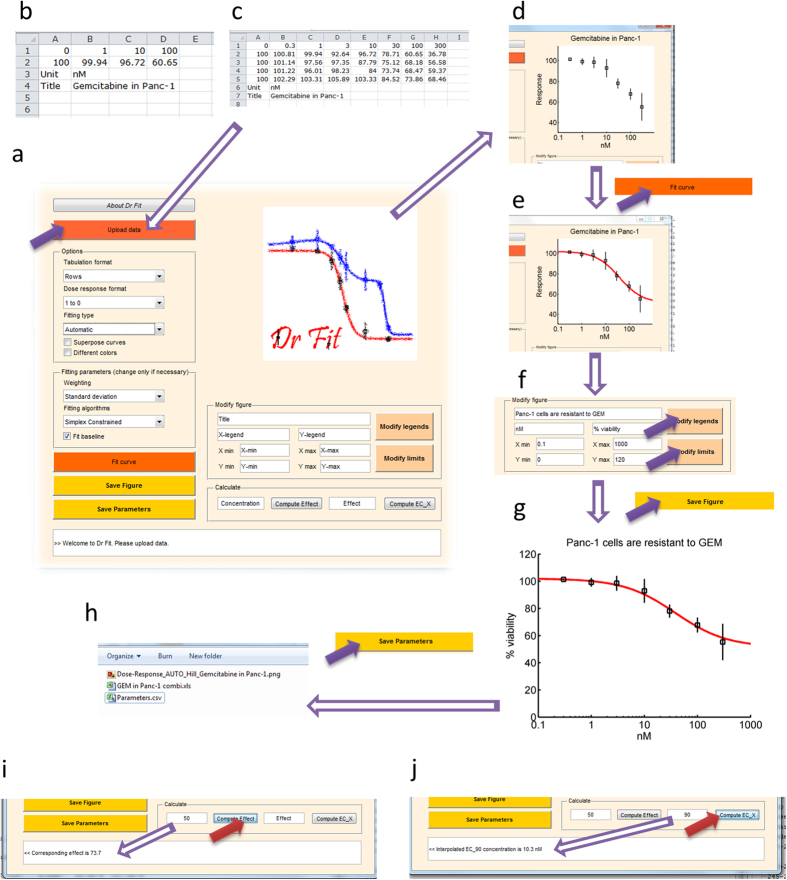
*Dr-Fit* software. *Dr-Fit* (Dose-response fitting) is freely available software which implements our model and algorithm. It enables fitting dose-response curves with complex multiphasic features. **(a)** User-friendly interface enables data access, option selection, running the algorithm and saving the results. **(b)** Experimental data is saved in .xls file (in this example one replicate and three concentration points). **(c)** Several replicates and various concentration points can be used (here the example has been expanded to four replicates and seven concentration points). **(d)** The uploaded data is displayed in the top-right panel. **(e**) Following the model-building process, the resulting curve is also displayed in the same panel. **(f)** The figure legend and limits can be modified through the centre-right panel. **(g)** The plot can then be automatically saved in high quality .png figures (resulting figure is shown) or accessed and saved in alternative formats. **(h)** Model’s parameters can also be saved in the project’s folder. **(i)** Effects can be interpolated or extrapolated via the bottom left panel. **(j)** Additionally, effective concentrations can be computed for any desired effect (e.g. EC_50_). Here the EC_90_ which is the effective concentration that gives a 90% effect has been calculated.

We developed an optimization process to automatically generate and provide models with either one phase of inhibition, two phases of inhibition, one stimulatory and one inhibitory phase, or one stimulatory and two inhibitory phases. Our approach was based on an optimization algorithm and a ranking test^40^ which were both incorporated in the Dr-Fit software (Fig. 4; http://sourceforge.net/projects/drfit/). Details of the overall method, including the formulation of the optimized objective function and how to use the software, can be found in the methods section.

**Figure 5:**
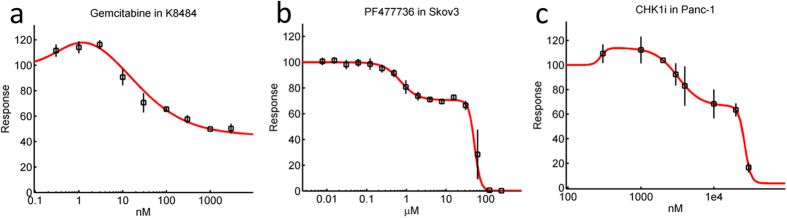
Modelling non-monophasic cases. Our new model and method enable to also describe experimental dose-responses which are not monophasic. **(a)** Our first (initial stimulatory effect), **(b)** second (two inhibitory phases) **(c)** and third example (initial stimulatory effect and two inhibitory phases) are well described by the resulting model in each case (red line).

Our approach was used to model the three examples of multiphasic dose-response curves shown in Fig. 2 and resulted in excellent descriptions of the data (Fig. 5). Each case was classified as a mono-phasic or multiphasic dose-response based on the Bayesian Information Criterion (BIC). The BIC criterion enables robust model ranking and selection and is based on a Bayesian approach^40^. The BIC criterion was chosen here over other classical ranking methods because it penalizes over-fitting more than other well-known ranking criteria^41^,^42^,^43^. Thus a conservative approach is used here where simpler models are favoured versus more complex multi-phasic ones.

Using this approach, the first case was recognized as a 2 processes model involving a stimulatory and an inhibitory phase (Fig. 5a). The second case was recognized as a 2 processes model involving two inhibitory phases (Fig. 5b). The third case was recognized as a 3 processes model involving a stimulatory and two inhibitory phases (Fig. 5c). We then wanted to assess what proportion of cases might be better described by a multiphasic dose-response curve rather than a standard Hill equation.

**Figure 6:**
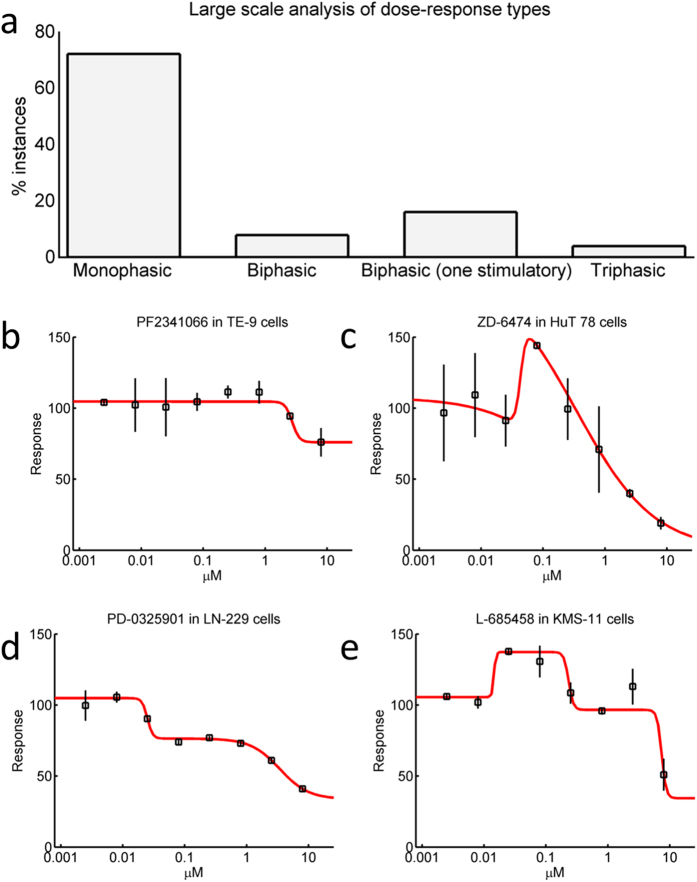
Large scale analysis of dose-response types. We analysed 11650 dose-response experimental cases available from the Cancer Cell Line Encyclopedia (CCLE). **(a)** Histogram showing the distributions of models which better described each case. **(b)** An example of monophasic case. **(c)** An example where an initial stimulatory phase is present (also termed hormesis). **(d)** An example where two inhibitory phases are present. **(e)** An example where both an initial stimulatory phase and two inhibitory phases are present.

As an example, we analysed 11650 dose-response experimental cases available from the Cancer Cell Line Encyclopedia (CCLE)^17^,^44^. This data-set was generated using High-Throughput Screening (HTS) technology and its analysis presents several challenges. First, the very large amount of data precludes visual inspection of each case. Second, the noisy nature of HTS generated data complicates model identification. Our approach was successful in analysing this data-set and showed that (Fig. 6a), based on our BIC ranking test, overall 72% of cases could be explained using a monophasic standard Hill equation (e.g. Fig. 6b). Thus, a substantial proportion of dose-responses were found to be better described by a multiphasic approach (28%). Most of these were appropriately described by a biphasic model with an initial stimulatory effect (16%, e.g. Fig. 6c). A substantial number (8%) was appropriately described with two point of inflection (e.g. Fig. 6d) and only 4% required a triphasic model (e.g. Fig. 6e).

## Discussion

Dose-response curves are routinely generated in the laboratory environment to assess the effect of drugs and other agents on molecular, cellular and other models. In most cases, a single inflection dose-response is observed which can be modelled using a classical Hill model. Nevertheless, there are instances where dose-responses show an initial stimulatory effect (also termed hormesis^20^) or is fully inhibitory but with two point of inflections (bi-phasic). In a number of instances, a combination of these features can be observed. It is often desirable to model the observed dose-response in order to obtain a mathematical description which can also be used to interpolate, extrapolate or derive metrics such as *EC*_*50*_ or maximum effects.

The challenge in identifying and modelling multi-phasic dose-response curves can be attributed to the increased complexity and decreased interpretability of potential approaches to model multiphasic dose-responses. Increased complexity is often associated to poor fitting or difficulty in generating appropriate fitting, particularly for experimentalists who are not necessarily familiar with these procedures. We have therefore developed a general model which we encapsulated in a fitting-ranking procedure and software, producing a robust, user-friendly tool.

**Figure 7:**
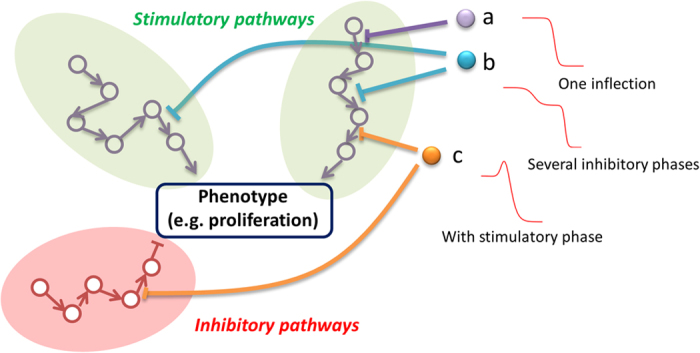
Mechanistic interpretation of our generalized Hill model. A specific phenotype (for instance cell proliferation) is often the result of several converging pathways. Some pathways result in stimulating a phenotype while others might inhibit it. **(a)** A drug may inhibit a specific node of a stimulatory pathway, resulting in a monophasic dose-response as per the classical Hill model. **(b)** In some cases a drug may affect several nodes, possibly in different stimulatory pathways and with different potency. If the difference in potency is great enough, this might lead to a clearly observable bi-phasic dose-response. **(c)** If the drug inhibits an inhibitory pathway at lower concentration, a stimulatory phase can also be observed.

Most advances in modelling dose-response data have focused on hormetic effects and have been made in the fields of toxicology and environmental science^28^,^45^,^46^,^47^,^48^,^49^. Our approach is based on simply combining the classical Hill and Bliss models and enables describing dose-response data with complex multiphasic features. Thus we explain observable inflections as the result of perturbing underlying independent processes (Fig. 7). Each Hill model term in our formulation can be interpreted as the effect of the drug interacting with a series of converging, cooperative processes^12^ (e.g. interaction with a specific biological pathway). The product of these Hill model terms can then be interpreted in terms of perturbed processes (e.g. pathways) which are independent^50^. Therefore our approach enables describing dose-response data in a robust manner and it also offers interpretation in broad mechanistic terms.

It should be noted that dose-response curves, including those with two or more inflections, can in theory be fitted using splines^51^,^52^ or other polynomials-based approaches^53^,^54^. The question therefore arises to why an alternative approach such as ours would be useful. We believe that the answer to this question is rooted in the same reasons which make the use of the classical Hill model popular for traditional monophasic dose-response curves. The first reason is that the number of parameters used with splines fitting increases linearly with the number of knots (the points where the polynomial pieces connect). This adversely affects the goodness of fit as measured via a maximum likelihood approach. The second reason is that when using splines, the resulting dose-response model is not indicative of potential underlying mechanisms. This is not the case in our approach where the resulting model structure indicates if there is a stimulatory effect or/and a second inhibitory phase (which could indicate interference with distinct processes). Also, the parameters do not correspond to specific features of the curve (steepness, half inhibitory concentration, maximum effect). In respect of all these points, our model is in line with the classical Hill model and can be viewed as an extension of it in the case of multiphasic cases.

We have also developed an algorithm which enables determination of how many processes can be observed (in order to tailor the model at the right level) based on statistical consideration. The resulting procedure has been integrated in our newly developed user-friendly Dr-Fit software. We also proceeded to analyse a large data-set and showed that, based on models ranking, a substantial number of cases do not fit the Hill model and might require multiphasic models (28%). It should be noted that several authors already highlighted the importance and implications of multiphasic dose-response such as hormetic effects^19^,^20^. More recently, the relevance of complex dose-response mechanisms has also been highlighted in the context of cancer therapeutics^21^,^22^. Our finding that a substantial proportion of a large data-set is better described by a multiphasic model might suggest that these effects are not exceptionally rare.

From a practical point of view, this approach enables classifying observed dose-response in a number of ways. It enables the user to distinguish between mono- vs. multi-phasic dose-response. It also enables the user to distinguish between various types of multi-phasic features (e.g. stimulatory + inhibitory phases vs. stimulatory + 2 inhibitory phases). Moreover, more quantitative comparisons across agents or systems can be performed using parameters such as the *EC*_*50*_ for each one of the identified phases. Parameters also provide mechanistic insight, for instance in terms of understanding at what concentration level a specific process is engaged (e.g. first inhibitory vs. second inhibitory phase).

Interpolation and extrapolation exercises are improved using this approach. This can have impact in a number of instances, such as for instance in the case of assessing drug combinations^55^. It should also be noted that other important metrics such as the area under the curve (AUC) can be derived from the dose-response model^56^. For instance, the AUC together with the IC50 were considered to compare the results of two large-scale pharmacogenomics studies^16^. The calculation of such metrics should also benefit from our new approach.

The implementation of our general model and method in Dr-Fit software should enable robust processing of both standard dose-response curves and multiphasic ones. We expect Dr-Fit to be a tool useful for many experimentalists and analysts interested in the study of agent effects on biological systems.

## Material and Methods

### Modelling

The model introduced in the Results section was implemented using Matlab. A three processes model was implemented using the following equation:


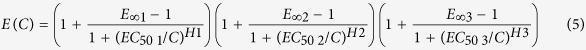


Dr-Fit software reads the dose-response data and builds the model. The algorithm implemented in Dr-Fit first attempts to fit the data to a one process model (i.e. a standard Hill equation). This is followed by an attempt to fit the data to a two inhibitory processes model. Then Dr-fit attempts to fit the data to a two processes model which includes a stimulatory process. Finally, the software attempts to fit the data to a three processes model, two of them being inhibitory (antagonist) and one stimulatory (agonist). Three optimization algorithms were implemented to find the model parameters: the simplex constrained, the Trust-region-reflective and the standard simplex. The Simplex constrained is the same algorithm as per the Simplex but constrains the parameters of the stimulatory and inhibitory processes such that no overlap in concentration space occurs (well separated processes). For all optimization algorithms, the following function *F* is minimized:


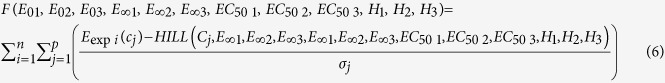


where *c*_*j*_ corresponds to the j^th^ concentration (*p* concentrations), *E*_*exp i*_*(c*_*j*_) corresponds to the measured effect for concentration level *c*_*j*_ and replicate *i* (*n* replicates), and *σ*_*j*_ is the standard deviation obtained for all the measures at concentration *c*_*j*_. For models with only two processes *EC*_*50 3*_, *H*_*3*_ and *E*_*∞3*_ were fixed to 1. For the model with only one process (i.e. a Hill model), *EC*_*50 2*_, *H*_*2*_ and *E*_*∞2*_ were also fixed to 1.

For normally distributed observational noise, minimizing our function F corresponds to a maximum likelihood estimate of the parameters. Thus, when more than one replicate is available, goodness of fit (GOF) can be tested for and is also provided by the software. The resulting four models are then ranked based on the Bayesian Information Criterion (BIC) and the model with the lowest BIC score is proposed by the software as the best model^40^. The BIC is our favourite ranking score because it favours simpler models, thus avoiding overparmetrization^41^,^42^,^43^ (the Akaike Information Criterion^57^ (AIC) score is also provided by the software if needed). In some instances, the absence of an appropriate number of replicates prevents satisfactory assessment of the standard deviation. In this case the software also enables weighting the sum of squares in the function *F* by unity rather than the standard deviation.

It is also important to note that, if we assume for instance that the dose-response data is normalized to control conditions (no drug), then measured effects vary from 1 at low concentration to a value often close to 0 at high concentration. In practice, it is possible that fluctuations lead to a poor estimation of control conditions (i.e. not enough replicates, spatial effects in plated experiments etc.) such that the baseline may appear greater or lower than 1. Thus, the option is given to also scale the model in order to capture a different baseline and minimize these effects.

### Software main features

Dr-Fit software was developed to enable end-users to easily model their dose-response data without requiring any coding or scripting. The software can be downloaded from sourceforge.net/projects/drfit and it is easily installed on windows-64 machines. Dr-Fit is accessible through an user-friendly interface (Fig. 4a). Experimental data is simply tabulated in a .xls file (Fig. 4b) and there are no limits in terms of concentration points and number of replicates (Fig. 4c; each replicate is simply added next to the previous one). The data for the software should be ordered as follows: concentrations, effects for replicate 1, effects for replicate 2 etc, then concentration unit and then title. The data can be ordered either in column or rows and several formats for the dose-response can be selected (Effects varying from 1 to 0 or 100 to 0 or 0 to 1 etc). A template and several examples are provided online.

Once the data is loaded, it is displayed in the right panel of the software (Fig. 4d). The user can then proceed to automatically produce a model which will be also displayed in the right panel. Upon pressing the “Fit curve” button, Dr-Fit proceeds to automatically fit the data (Fig. 4e). The plots can be further modified (Fig. 4f) and saved as high quality figures (Fig. 4g). The models’ characteristics (parameters, scaling, χ^2^, GOF, AIC and BIC) can also be saved in a .xls file in the project’s folder (Fig. 4h). Additionally, effects can be interpolated or extrapolated at various concentrations (Fig. 4i) and effective concentration at 50% (i.e. EC_50_ of the overall) or at any other level (e.g. EC_90_) can be calculated via Dr-Fit interface (Fig. 4j). Several dose-response curves can be displayed in the right panel (with same or different colour) and the whole figure saved with high resolution.

### Cells and reagents

The KPC K8484 cancer cell line was established from a KPC PDAC tumour^58^. PANC-1 cells were obtained from the European Collection of Cell Cultures (ECACC; Health Protection Agency, Salisbury, UK) and were verified by STR genotyping and tested negative for mycoplasma. Cells were grown in DMEM medium (Life Technologies) supplemented with 5% fetal calf serum at 37 °C and 5% CO2. SKOV3 cells were obtained from ATCC and tested negative for mycoplasma. They were grown in RPMI medium (Life Technologies) supplemented with 5% fetal calf serum at 37 °C and 5% CO2. Gemcitabine was obtained from Tocris Bioscience (Bristol, UK).

Gemcitabine was dissolved in dimethylsulphoxide (DMSO) and then diluted in culture medium to a final concentration of 0.2% DMSO. PF477736 was obtained from SynKinase (Parkville, Austrailia), dissolved in DMSO at 10 mM, and serially diluted to the appropriate concentration.

### Cytotoxicity assay

For K8484 and PANC-1, drug cytotoxicity *in vitro* was assessed by the means of Sulforhodamine B colorimetric (SRB) assay. Cells were plated with a range of concentrations. After 72 h of incubation at 37 °C, they were fixed (3% trichloroacetic acid in water (w/v), 90 minutes, 4 °C), washed in water and stained with a 0.057% SRB (Sigma) solution in acetic acid (w/v) for 30 minutes. The plates were washed (1% acetic acid (v/v), 4 times), and the protein-bound dye was dissolved in a 10 mM Tris base solution (pH 10.5). Fluorescence was measured using Tecan Infinite M200 plate-reader (excitation 488 nm, emission 585 nm). Percentage inhibition compared to solvent control-treated cells was calculated for each drug concentration.

For SKOV3, drug toxicity was assessed by cell titer glo. Approximately 500 cells per well in 5 uL was dispensed using a Multidrop Combi dispenser (Thermo Fisher Scientific) into 1,536 solid-bottom white Greiner Bio-one tissue culture-treated plates (catalog #789173-F). 23 nL of ). PF477736 was transferred to the assay plate using a Kalypsis pintool. Plates were covered with stainless steel cell culture lids and incubated at standard conditions for 48 hours. To assess viability, 3 uL of CellTiter Glo luminescent cell viability assay reagent (Promega) was added using a Bioraptor Flying Reagent Dispenser (Aurora Discovery-BD). The plates were incubated for 15 minutes at room temperature and measured using a 10-s exposure on a ViewLux (Perkin-Elmer).

## Additional Information

**How to cite this article**: Di Veroli, G.Y. *et al.* An automated fitting procedure and software for dose-response curves with multiphasic features. *Sci. Rep.* **5**, 14701; doi: 10.1038/srep14701 (2015).
